# Identification of copper death-associated molecular clusters and immunological profiles in rheumatoid arthritis

**DOI:** 10.3389/fimmu.2023.1103509

**Published:** 2023-02-20

**Authors:** Yu Zhou, Xin Li, Liqi Ng, Qing Zhao, Wentao Guo, Jinhua Hu, Jinghong Zhong, Wenlong Su, Chaozong Liu, Songchuan Su

**Affiliations:** ^1^College of Traditional Chinese Medicine, Changchun University of Chinese Medicine, Changchun, China; ^2^Foot & Ankle Surgery, Chongqing Orthopedic Hospital of Traditional Chinese Medicine, Chongqing, China; ^3^Jilin Ginseng Academy, Changchun University of Chinese Medicine, Changchun, China; ^4^Institute of Orthopaedic and Musculoskeletal Science, University College London, Royal National Orthopaedic Hospital, London, United Kingdom; ^5^School of Health Management, Tianjin University of Chinese Medicine, Tianjin, China; ^6^College of Pharmacy, Changchun University of Chinese Medicine, Changchun, China

**Keywords:** rheumatoid arthritis, copper death, machine learning, immune infiltration, predictive models

## Abstract

**Objective:**

An analysis of the relationship between rheumatoid arthritis (RA) and copper death-related genes (CRG) was explored based on the GEO dataset.

**Methods:**

Based on the differential gene expression profiles in the GSE93272 dataset, their relationship to CRG and immune signature were analysed. Using 232 RA samples, molecular clusters with CRG were delineated and analysed for expression and immune infiltration. Genes specific to the CRGcluster were identified by the WGCNA algorithm. Four machine learning models were then built and validated after selecting the optimal model to obtain the significant predicted genes, and validated by constructing RA rat models.

**Results:**

The location of the 13 CRGs on the chromosome was determined and, except for GCSH. LIPT1, FDX1, DLD, DBT, LIAS and ATP7A were expressed at significantly higher levels in RA samples than in non-RA, and DLST was significantly lower. RA samples were significantly expressed in immune cells such as B cells memory and differentially expressed genes such as LIPT1 were also strongly associated with the presence of immune infiltration. Two copper death-related molecular clusters were identified in RA samples. A higher level of immune infiltration and expression of CRGcluster C2 was found in the RA population. There were 314 crossover genes between the 2 molecular clusters, which were further divided into two molecular clusters. A significant difference in immune infiltration and expression levels was found between the two. Based on the five genes obtained from the RF model (AUC = 0.843), the Nomogram model, calibration curve and DCA also demonstrated their accuracy in predicting RA subtypes. The expression levels of the five genes were significantly higher in RA samples than in non-RA, and the ROC curves demonstrated their better predictive effect. Identification of predictive genes by RA animal model experiments was also confirmed.

**Conclusion:**

This study provides some insight into the correlation between rheumatoid arthritis and copper mortality, as well as a predictive model that is expected to support the development of targeted treatment options in the future.

## 1 Introduction

Rheumatoid arthritis (RA) is a chronic inflammatory joint disease where patients usually exhibit symptoms such as synovitis and bone erosion induced by cartilage destruction and osteoclast activation. This eventually destroys the patient’s bone, cartilage and tendons, and in severe cases results in deformity and disability which seriously affects the patient’s quality of life (1–4). In addition, if patients do not receive timely and effective treatment after the onset of the disease, it may lead to a series of complications such as cardiovascular disease, making it more difficult to treat clinically (5, 6). Studies have shown that about 1% of the world’s population is affected by RA, which can occur in any age group and is about 2-3 times more common in women than in men (7). The pathogenesis of RA is still unclear, but it is clinically believed that there is a close relationship between genetic susceptibility, environmental factors and the development of the disease (8). Notably, RA is affected by the infiltration of multiple inflammatory cells and the release of abnormal cytokines. A range of immune cells, including macrophages, dendritic cells, mast cells, neutrophils, T cells and B cells are all closely associated with RA (9, 10).

The mitochondrion is a semi-autonomous organelle with a double membrane structure, which can provide cell energy through the tricarboxylic acid cycle (TCA) oxidative phosphorylation (11, 12). Previous studies have found that imbalances in mitochondrial homeostasis may contribute to the development of various autoimmune diseases including RA, scleroderma, desiccation syndrome and systemic lupus erythematosus (13, 14). Thus, there is a relationship between RA and mitochondria. Copper death is a new mode of cell death that is distinct from iron death and necroptosis, and its related genes include SLC31A1, PDHB, PDHA1, LIPT1, FDX1, DLD, DLST, DBT, LIAS, DLAT, GCSH, ATP7A and ATP7B (15, 16). The main mechanisms of copper death are the excessive accumulation of lipoylated mitochondrial enzymes and depletion of Fe-S cluster proteins (17, 18). Notably, copper ions (Cu^2+^) act as co-factors for the enzyme, and copper homeostasis is closely related to the presence of mitochondrial regulation (19). Copper is mainly present in the form of cytochrome C oxidase (COX) and superoxide dismutase (SOD1) in mitochondria and plays an important regulatory role in the TCA process, ultimately interfering with various biological processes such as redox homeostasis, iron utilisation, oxidative phosphorylation and cell growth (20, 21). An analysis of 1444 patients with RA showed that serum levels of copper were significantly higher in RA patients compared to the healthy population (22). It was also suggested that when the human synovial membrane is exposed to hypoxic conditions or excessive glycolysis in multiple cells, copper death may be inhibited, leading to excessive survival and proliferation of various immune cells such as fibroblast-like synoviocytes, effector T cells and macrophages, further leading to inflammatory responses and bone destruction in RA patients; Meanwhile, important regulatory genes of copper apoptosis such as PDHA1, DLAT, FDX1MTF1 and LIAS have been shown to be closely associated with the RA process (23). Therefore, we hypothesise that there may be a relationship between the onset and progression of rheumatoid arthritis and copper death.

The present study analysed for the first time the differential expression and immune profile of CRG between RA and non-RA individuals. We divided 232 RA patients into two copper death-related molecular clusters based on 13 CRG expression profiles, and further analysed expression differences and immune cell differences between the two. RA patients were further grouped according to the genes that subsequently intersected between molecular clusters and gene clusters, their expression profile and immune profile were analysed, along with their copper death-related scores. The differentially expressed genes (DEGs) in the GSE93272 data were obtained, the specific genes between CRGclusters were identified by the WGCNA algorithm, and an optimal prediction model was built using four machine learning methods. The performance of these prediction models was then validated by the Nomogram model, Calibration curve and DCA, and the expression profiles of five important genes obtained from the optimal prediction models were also demonstrated.

## 2 Methods

### 2.1 RA and copper death-related gene data collection and analysis

Retrieval of RA microarray data from Gene Expression Omnibus (GEO) (www.ncbi.nlm.nih.gov/geo) (24, 25). The test set GSE93272 was reported by Tasaki S (15) et al. which provides transcriptomic data from whole blood obtained from a population of RA patients (n = 232) and healthy controls (n = 43) from the GPL570 ([HG-U133_Plus_2] Affymetrix Human Genome U133 Plus 2.0 Array). The expression matrix of the experimental and control samples in GSE93272 was obtained using a Perl (V5.35.0), and the differential genes between samples in the dataset were obtained using the R language. The source of copper death-related genes was Tsvetkov et al (15). A total of 13 CRGs were obtained.

### 2.2 Correlation between CRG and immune cells and chromosomal analysis

To further demonstrate the association between CRG and RA-related immune cell properties, we analysed the correlation coefficient between CRG expression and the relative percentage of immune cells using the “corrplot” R package. The location of the CRG on the chromosome was obtained using the perl program, the “RCircos” package in the R language.

### 2.3 Assessment of immune cell infiltration

The relative abundance of each sample in 22 immune cells was assessed based on the gene expression data of GSE93272 using the CIBERSORT algorithm (26). p<0.05 represents a significant difference.

### 2.4 Unsupervised clustering and gene set variance analysis of RA patients

The “ConsensusClusterPlus” package (27) was used to perform unsupervised cluster analysis by classifying 232 RA samples into different clusters using the k-means algorithm for 1000 iterations. The maximum number of clusters k = 9 and the optimal number of clusters were synthesised based on the cumulative distribution function (CDF) curve, consensus matrix, and consistent cluster score. PCA was then used in conjunction with t-distributed stochastic neighbour embedding (tSNE) to determine whether these genes could be used to distinguish samples (28, 29).

### 2.5 Re-consensus clustering based on CRGcluster intersection genes

Based on the intersecting genes obtained from the unsupervised clustering of RA patients, we used the ConsensusClusterPlus and limma packages to reclassify the RA patients and identify different subgroups (30). Cluster-to-cluster expression and immune profiles were analysed. The expression of CRG between CRGcluster and genecluster clusters was analysed using the “ggpubr” and “reshape2” packages.

### 2.6 Differential gene enrichment analysis and weighted gene co-expression network analysis

We used the R language to screen the DEGs in the GSE93272 dataset according to Padj < 0.05 and ∣logFC∣ > 0.5, and the results are presented in the form of volcano plots and heat maps. In addition, we performed GO and KEGG enrichment analysis on the above 276 DEGs using the R language. We used the ‘WGCNA’ package (31) in the R language to identify co-expression modules. The top 25% of genes with the highest variability were applied to the subsequent WGCNA analysis to ensure the accuracy of high quality results. We selected the best soft threshold to construct a weighted neighbour-joining matrix and further converted it into a topological overlap matrix (TOM). Modules were obtained using a TOM dissimilarity measure (1-TOM) based on a hierarchical clustering tree algorithm when the minimum module size was set to 100. Each module is assigned a random colour. The module signature genes represent the global gene expression profile in each module. The relationship between module and disease state is expressed through modular significance (MS), and gene significance (GS) is described as the correlation between a gene and a clinical phenotype (32).

### 2.7 Building predictive models based on multiple machine learning methods

The Random forest model (RF) is an integrated machine learning method that can use multiple independent decision trees for classification or regression (33, 34). The support vector machine model (SVM) can generate hyperplanes with maximum margins in the feature space to effectively differentiate data points (35). The generalised linear model (GLM) is based on and extends the multiple linear regression model to provide a more efficient and flexible assessment of the relationship between normally distributed dependent features and categorical or continuous independent features (36). eXtreme Gradient Boosting (XGB) is a collection of boosted trees based on gradient boosting, which is an effective algorithm to compare classification error and model complexity (37). We’ve used the ‘caret’ package in R to build a machine learning model based on 2 different CRG clusters (including RD, SVM, GLM and XGB). The 232 RA samples were randomly divided into training set (70%, N = 163) and validation set (30%, N = 49). All machine learning models were executed according to default parameters and evaluated in a comprehensive manner using 5-fold cross-validation. The four machine learning models are interpreted and their residual distribution and feature importance are visualised using the ‘DALEX’ package in R. The “pROC” R package was used to visualise the area under the ROC (receiver operating characteristic) curve (Area Under Curve, AUC). The best machine learning model was determined, and the top five genes included were used as predictors for RA.

### 2.8 Construction and validation of the nomogram model

A Nomogram model was constructed based on the predictor genes obtained from the RF, and the RA cluster was evaluated using the “rms” R package (38). Each predictor variable is assigned a score and the ‘total score’ represents the sum of the scores of the above predictors. The calibration curve and DCA were used to assess the predictive power of the Nomo model.

### 2.9 RF candidate gene expression diagnostic analysis

ROC curves were constructed to assess the diagnostic specificity and sensitivity of RF candidate genes, and the expression of each gene in non-RA samples and RA samples were analysed in the form of box plots.

### 2.10 Verification of the animal experiment

#### 2.10.1 Animals and models

10 male SD (6 weeks old, 140g each, SPF class) rats (n = 5 in each group) were obtained from Liaoning Changsheng Biotechnology Co. The experimental animal certificate number is SCXK (Liaoning) 2020-0001. The Institutional Animal Care and Use Committee of Changchun University of Traditional Chinese Medicine approved all experimental protocols and all experiments were conducted in accordance with relevant guidelines and regulations (No. ccucm-2017-0015). Animals were given one week to acclimatise to their new environment prior to the experiments. Each rat was randomly assigned to one of two groups with ten rats in each: the control group and the RA model group. The RA model group used a bovine type II collagen solution (concentration 2 mg/ml) added dropwise to an equal volume of incomplete Freund’s adjuvant to a final concentration of 1 mg/ml. The mixture was emulsified in an ice bath with a homogeniser and injected intradermally into the tail root at 0.2 mL per rat, followed by a booster immunisation at 0.1 mL per rat 7 days later. During the moulding period, the rats were subjected to wind, cold and humidity stimulation (wind speed 5m/s, humidity 90%-95%, temperature 0-2°C) for 14 days, once a day for 4 hours. The control group was injected intradermally with an equal volume of saline (39, 40). At the end of 14 days of moulding, the right ankle joint of the rat was taken for follow-up experiments.

#### 2.10.2 Quantitative reverse transcription-polymerase chain reaction

The expression levels of the DEGs were further verified by qRT-PCR (Applied biosystems, USA). Relative gene expression levels were normalised to Actin according to the 2-^ΔΔ^CT method. the primer sequences used are shown in **Table 1**.

**Table 1:** The primers used in this study.

Gene	Forward primer	Reverse primer
FAM96A	GGCAACTCTTATTGGACTGTG	TCGCTCTTTGTCATTTATCTGCT
MAK4P3	ATCCAGCAGGAAATTGTCA	TTCTACCTTGCATCCCGTG
PRPF39	GCAGCTTTTGAGGAACAAC	CATTGCCAATCCTAGAACAC
SLC35A1	AAAGAGTTCCGACACTTCC	CCAGACATAATACGTGTAGCC
TMX1	CCTGGTGTCCTGCTTGTCA	TAAACCGTCCACTTAGTCCTGT
Actin	CTGAACGTGAAATTGTCCGAGA	TTGCCAATGGTGATGACCTG

## 3 Results

### 3.1 CRG identification and immunoassay

**Figure 1:**
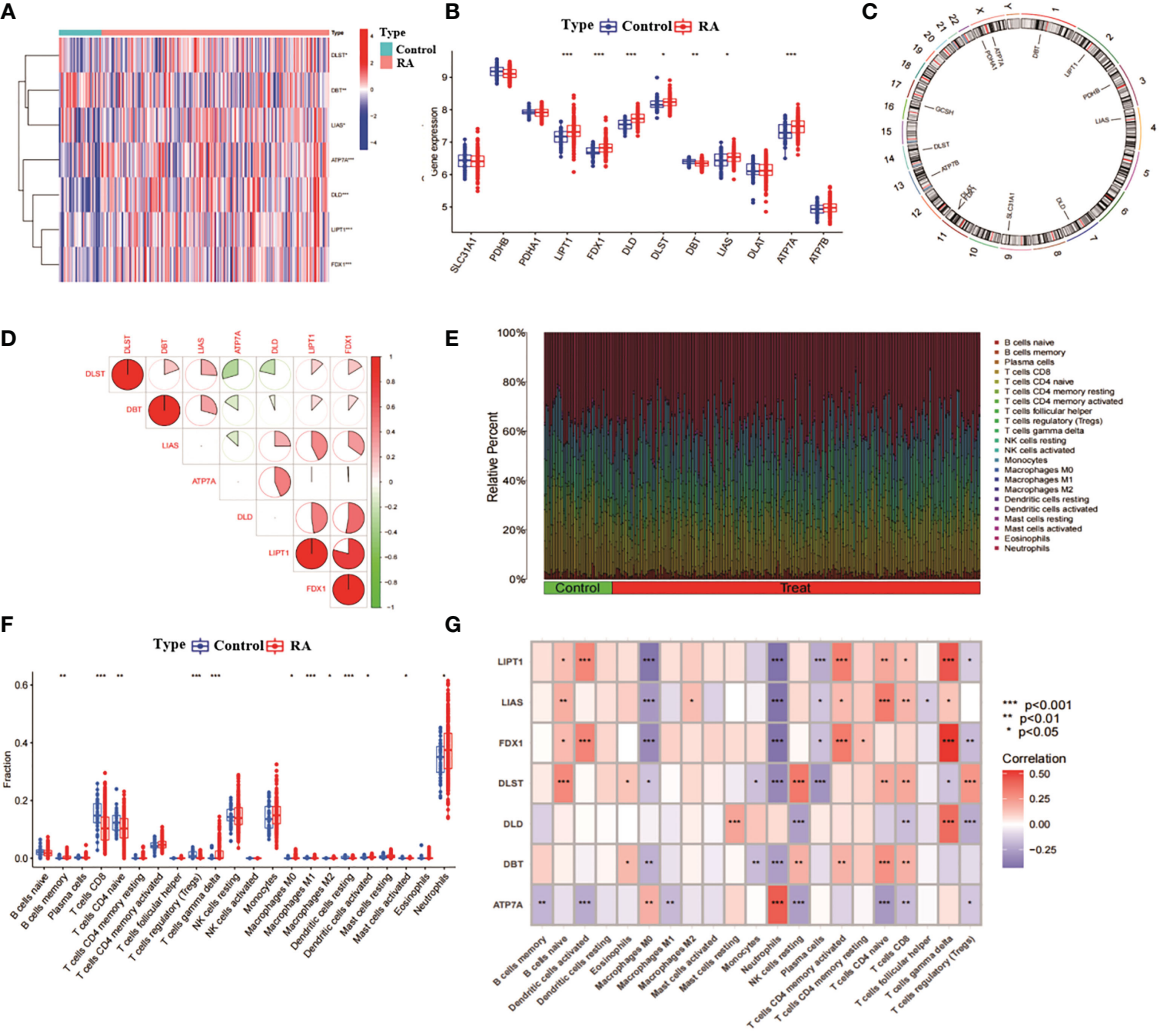
Correlation analysis of RA and CRG. **(A)** Heat map of 7 CRGs with differential expression. **(B)** Box line plot of expression of 12 CRGs in RA and non-RA. ***p< 0.0001, **p< 0.001, *p< 0.01, ns, not significant. **(C)** position of the 12 CRGs on the chromosome. **(D)** correlation analysis of the 7 differentially expressed CRGs, red and blue represent positive and negative correlations, respectively. The area of the pie chart indicates the correlation coefficient. **(E)** relative abundance of RA and non-RA among 22 infiltrating immune cells. **(F)** box plot of the differences in immune infiltration between RA and non-RA. **(G)** correlation analysis between 7 differentially expressed CRGs and infiltrating immune cells.

We analysed the expression of 13 CRGs between RA and non-RA controls through the GSE93272 dataset (of which GCSH was not present in this dataset, thus leaving 12 CRGs). The results identified seven CRGs as differentially expressed copper death genes. Of these, LIPT1, FDX1, DLD, DBT, LIAS and ATP7A were expressed at significantly higher levels in RA than non-RA levels, while DLST expression levels were significantly lower in RA than non-RA levels (**Figures 1A, B**). Subsequently, we analysed the location of the 12 CRGs on the chromosome (**Figure 1C**). We analysed the correlation between CRGs with differential expression to explore whether copper death genes play a role in the development of RA disease (**Figure 1D**). In addition, we performed an immune infiltration analysis between RA and non-RA samples (**Figure 1E**). The results showed that RA patients showed higher levels of infiltration in B cells memory, T cells CD8, T cells gamma delta, Macrophages M0, Macrophages M1, Macrophages M2, Dendritic cells activated, Neutrophils showed high levels of infiltration (**Figure 1F**). This suggests a close association between the development of RA and the immune system. Subsequently, we performed immune infiltration analysis on seven differentially expressed CRGs (**Figure 1G**). The results showed that there were significant positive correlations between LIPT1, FDX1, DLD and T cells gamma delta; LIAS, DLST, LIPT1, FDX1 and B cells naive; LIAS, LIPT1, DBT, DLST and T cells CD4 naive also had significant positive correlations and so on. It can be seen that CRG may play a key role in the regulation of RA and immune infiltration.

### 3.2 RA unsupervised clustering identification and analysis

**Figure 2:**
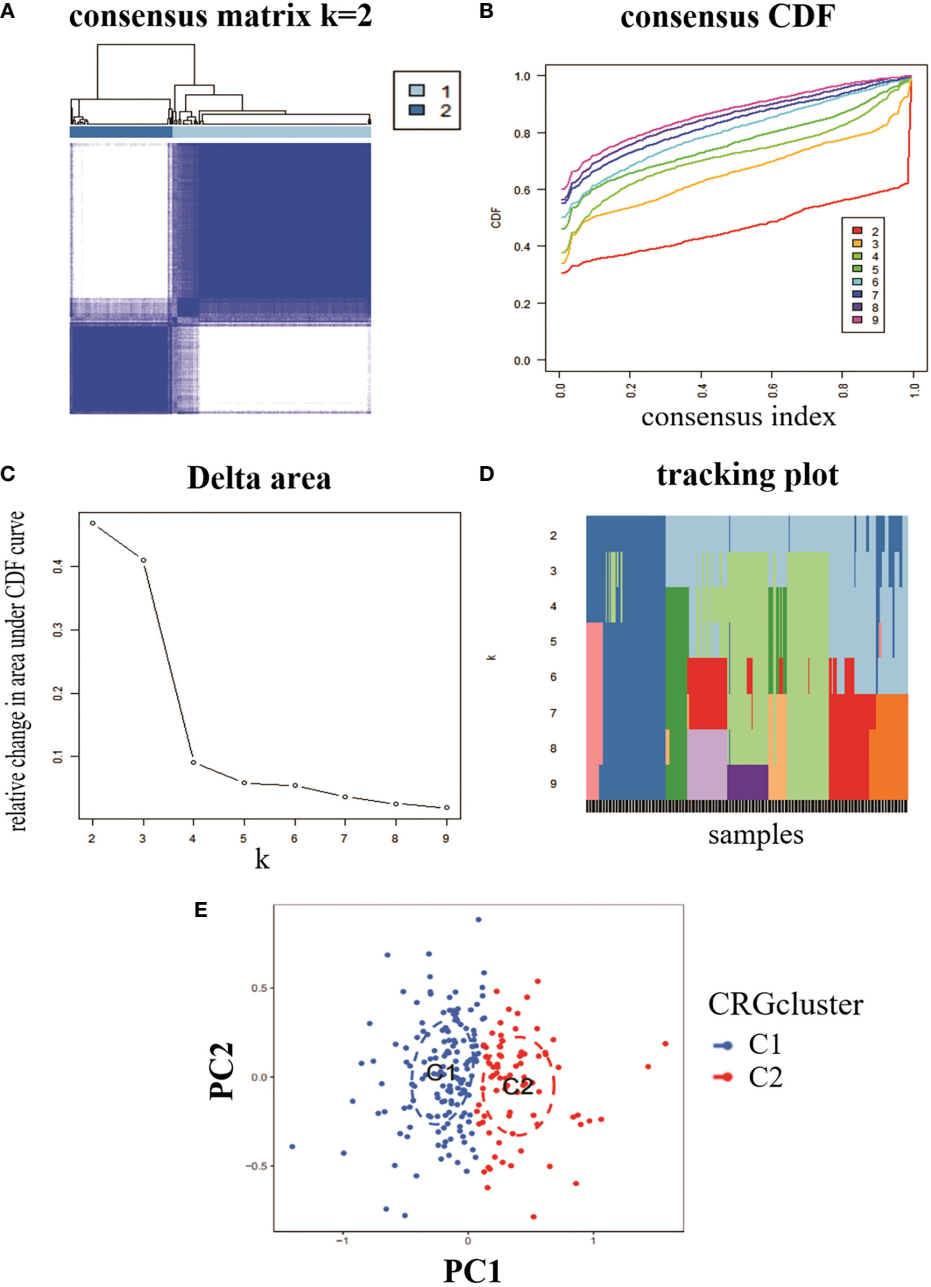
Identification of molecular clusters of genes associated with copper death in RA. **(A)** consensus clustering matrix at k = 2. **(B-D)** representative cumulative distribution function (CDF) curves, CDF incremental area curves, consensus clustering scores. **(E)** visual analysis of the two clustering distributions.

To analyse the expression of RA and CRG, we used a consensus clustering algorithm to identify 232 RA samples in groups with the expression profile of seven CRGs, with the best number of clusters when k = 2 (**Figure 2A**). The CDF values gradually increased when k = 2, 3 and 4, and became smaller when k = 4 (**Figures 2B–D**). We divided the 232 RA samples into two groups, including cluster 1 (n = 153) and cluster 2 (n = 79). PCA analysis of the two clusters showed significant differences between the two (**Figure 2E**).

### 3.3 Expression profile and immune infiltration characteristics of CRGcluster

**Figure 3:**
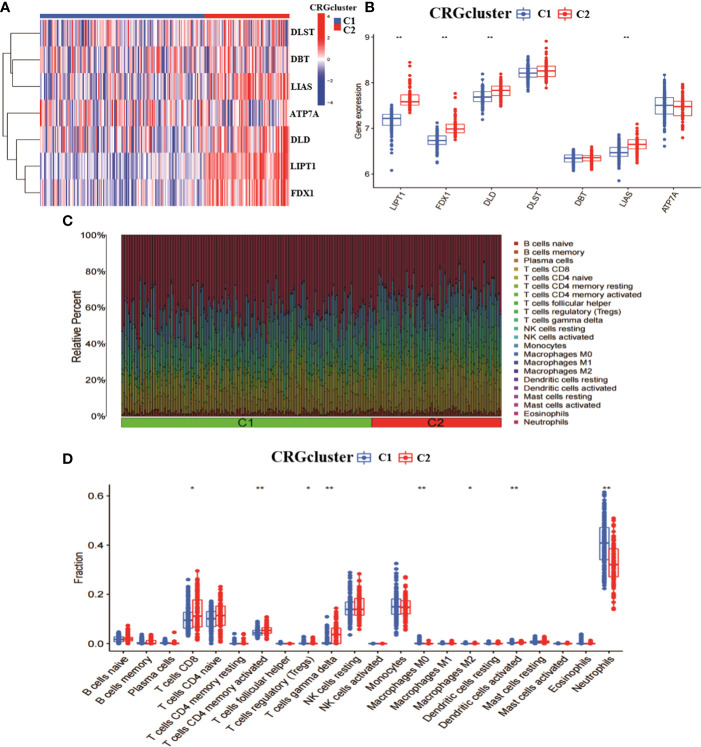
Analysis of the expression and immunological profile between the two molecular clusters. **(A)** Heat map of the expression of 7 CRGs between two molecular clusters. **(B)** Box line plot of the expression of 7 CRGs between two molecular clusters. **(C)** relative abundance between two molecular clusters in 22 infiltrating immune cells. **(D)** box line plot of immune infiltration between two molecular clusters. **p < 0.0001, *p < 0.01, ns, not significant.

To explore the characteristics among CRGclusters, we analysed the expression differences of seven CRGs between CRGcluster C1, and CRGcluster C2 (**Figure 3A**). CRGcluster C2 showed significant high expression levels of FDX1, DLD, LIPT1, and LIAS (**Figure 3B**). In addition, the immune infiltration of CRGcluster C1 and CRGcluster C2 was analysed (**Figure 3C**). The results showed that CRGcluster C2 showed higher levels in T cells CD8, T cells CD4 memory activated, and T cells gamma delta. CRGcluster C1 showed higher levels in T cells regulatory (Tregs), Macrophages M0, Macrophages M2, Dendritic cells activated, and Neutrophils had higher expression levels (**Figure 3D**). Taken together, this suggests that CRGcluster C2 may be more closely associated with the development of RA.

### 3.4 Consensus clustering analysis of CRGcluster intersection genes

**Figure 4:**
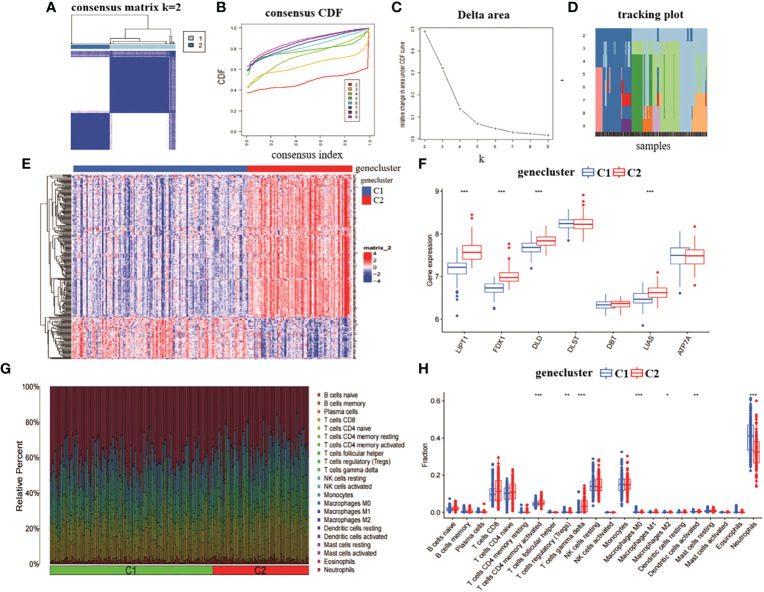
Identification of gene clusters and expression immunoassays in RA. **(A)** consensus clustering matrix at k = 2. **(B-D)** representative CDF curves, CDF incremental area curves, consensus clustering scores. **(E)** heat map of expression levels between two gene clusters. **(F)** box line plot of expression of 7 CRGs between two gene clusters. ***p< 0.0001, **p< 0.001, *p< 0.01, ns, not significant. **(G)** relative abundance between two molecular clusters in 22 infiltrating immune cells. **(H)** box line plot of immune infiltration between two gene clusters.

We analysed the role of the 314 intersecting genes in RA by re-clustering the intersecting genes obtained from CRGcluster C1 and CRGcluster C2 using “ConsensusClusterPlus” and further classifying RA patients into different subgroups. The number of clusters was optimal when k = 2 (**Figure 4A**). When k = 4, the CDF values became smaller (**Figures 4B–D**). We reclassified the 232 RA samples into two groups: genecluster 1 (n = 146) and genecluster 2 (n = 86).

We also analysed the expression differences of the seven CRGs between genecluster C1 and CRGcluster C2 (**Figure 4E**). genecluster C2 showed significantly higher expression levels of LIPT1, FDX1, DLD, and LIAS than genecluster C1 (**Figure 4F**). In addition, we analysed the immune infiltration of genecluster C1 and genecluster C2 (**Figure 4G**). The results showed that genecluster C2 had higher levels of T cells CD4 memory activated, T cells gamma delta. genecluster C1 had higher levels of T cells regulatory (Tregs), Macrophages M0, Macrophages M2, Dendritic cells activated, and Neutrophils (**Figure 4H**). Taken together, the correlation between genecluster C2 and RA may be higher than that between genecluster C1.

**Figure 5:**
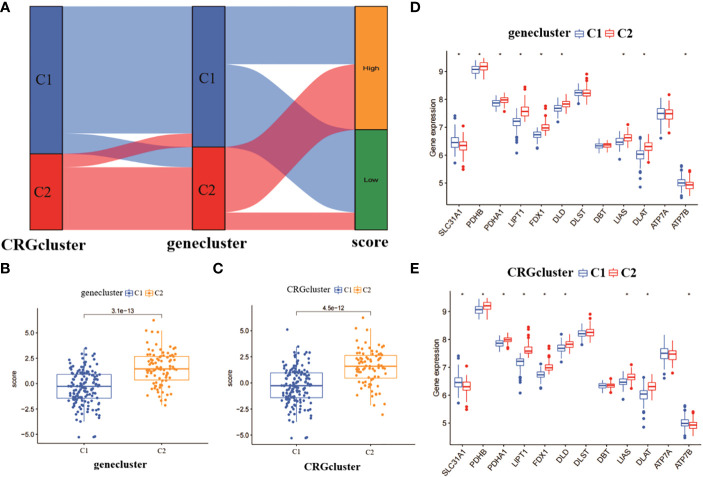
Analysis of copper mortality-related scores and expression differences. **(A)** Analysis of copper death correlation scores for molecular clusters and gene clusters. **(B)** Comparison of copper death scores between gene clusters. **(C)** Comparison of copper death scores between molecular clusters. **(D)** Differences in expression levels of 12 CRGs between gene clusters. **(E)** Differences in expression levels of 12 CRGs between molecular clusters *p< 0.0001.

We plotted the alluvial distribution of copper death-related score subtypes for CRGcluster C1, C2 and genecluster C1, C2 (**Figure 5A**). We compared the copper death-related scores between genecluster C1 and C2. The results showed that genecluster C2 had a significantly higher copper death-related score than genecluster C1 (**Figure 5B**). In addition, we also analysed the copper mortality-related scores between CRGcluster C1 and C2. The results showed that CRGcluster C2 had a significantly higher copper death-related score than CRGcluster C1 (**Figure 5C**). It can be seen that genecluster C2 and CRGcluster C2 correlated more significantly with copper death-related genes. We also compared the expression of 12 CRGs between CRGcluster C1, and C2 and genecluster C1, and C2. The results showed that PDHB, PDHA1, LIPT1, FDX1, DLD, LIAS and DLAT were significantly more expressed in CRGcluster C2 and genecluster C2 than in CRGcluster C1 and genecluster C1; while SLC31A1 and ATP7B were significantly more expressed in CRGcluster C1 and genecluster C1 than in CRGcluster C2 and genecluster C2 (**Figures 5D, E**).

### 3.5 Gene module screening and co-expression network construction

**Figure 6:**
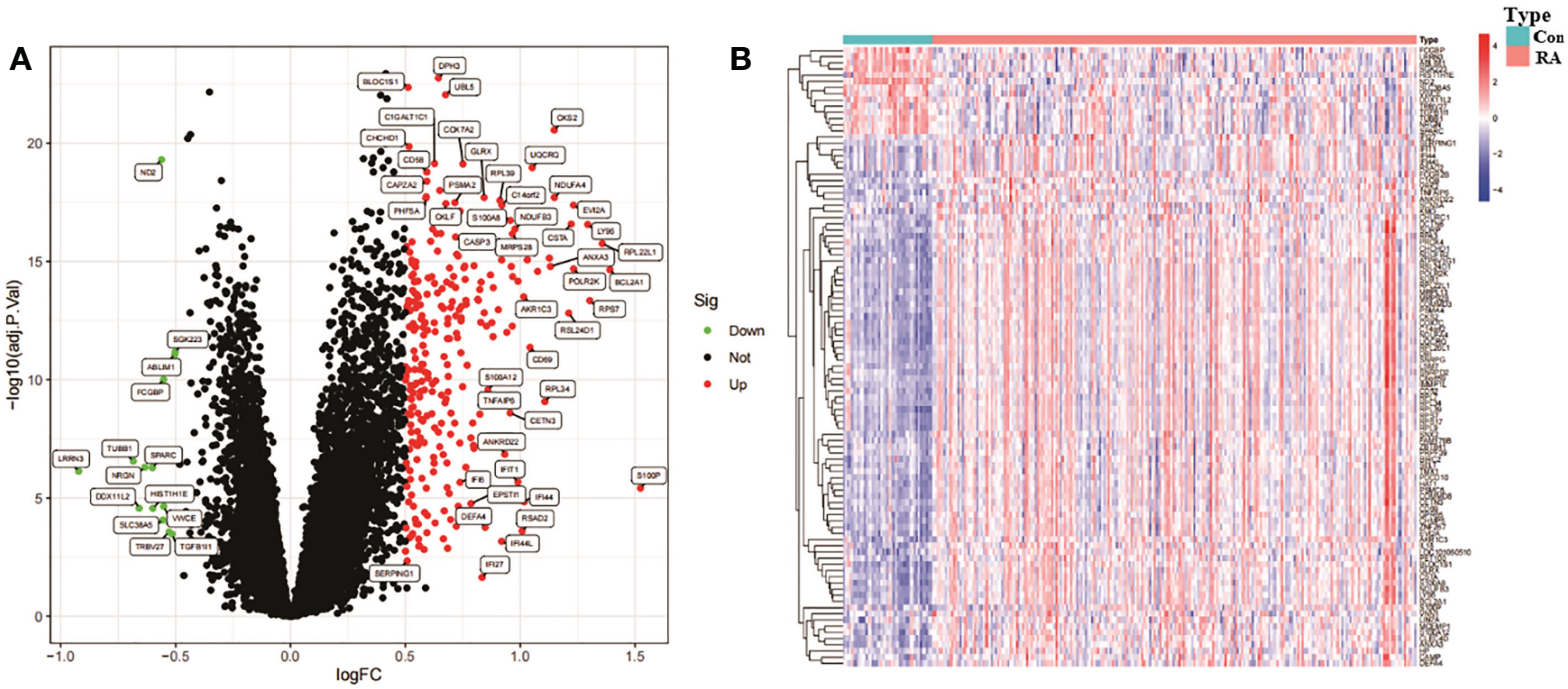
Identification analysis of DEGs. **(A)** DEGs volcano map. **(B)** DEGs heat map.

**Figure 7:**
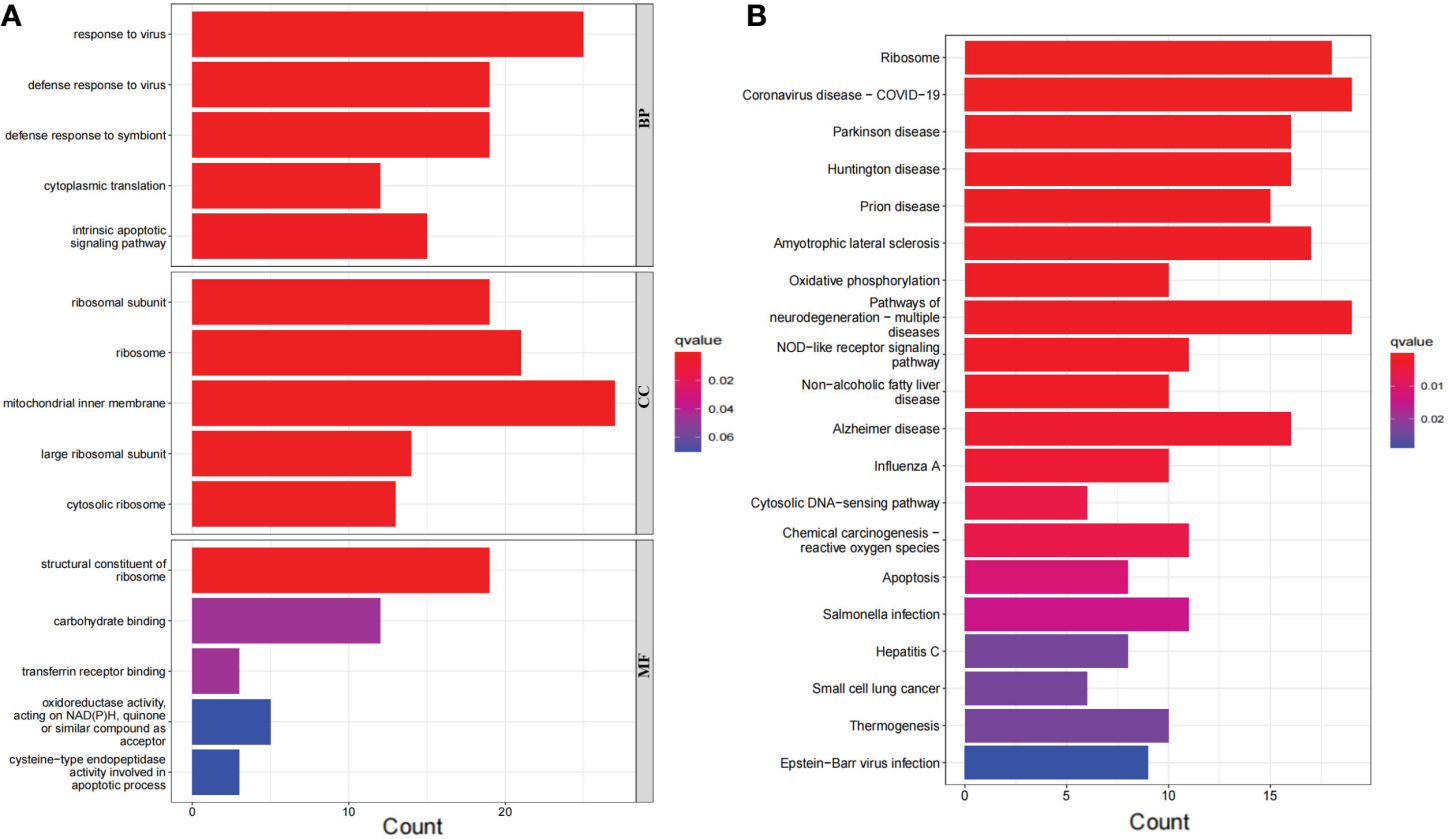
Bar graph of DEGs enrichment. **(A)** Bar chart of DEGs GO enrichment analysis. **(B)** Bar chart of DEGs KEGG enrichment analysis.

The 276 DEGs in GSE93272 were screened according to the criteria of Padj < 0.05 and ∣logFC∣ > 0.5 and are presented as volcano and heat maps (**Figures 6A, B**). In addition, we performed GO and KEGG enrichment analysis on the above 276 DEGs using R language (**Figures 7A, B**). The enrichment results showed that the 276 DEGs were mainly enriched in response to the virus, defence response to the virus and other pathways involved in Ribosome and Parkinson’s disease.

**Figure 8:**
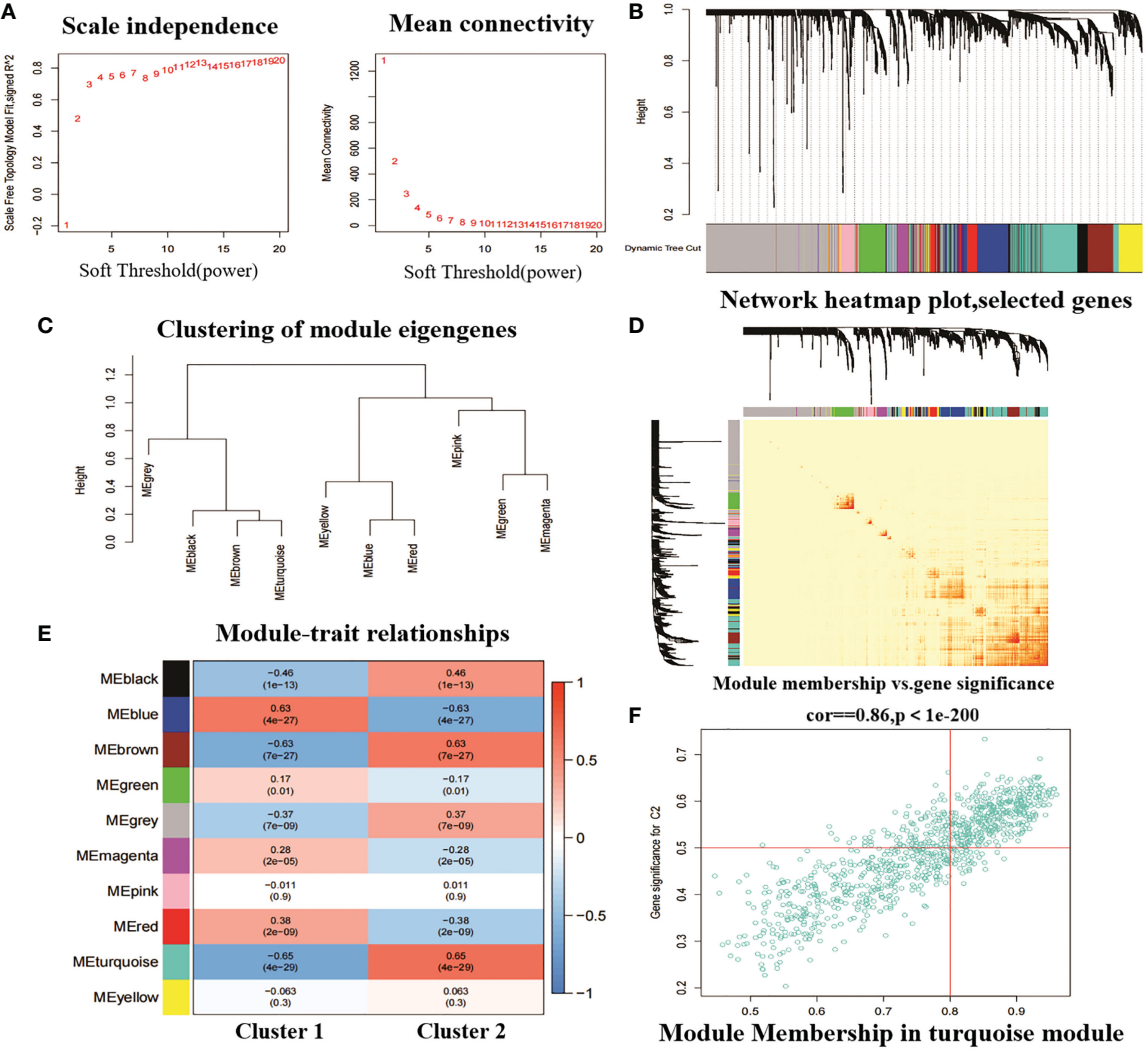
Weighted network analysis between two molecular clusters. **(A)** Determination of soft threshold power. **(B)** Cluster tree dendrogram of co-expression modules. Different colours indicate different co-expression modules. **(C)** representation of clusters of module signature genes. **(D)** representative heat map of correlations between the 10 modules. **(E)** correlation analysis between module signature genes and clinical status. **(F)** scatter plot between MEturquoise module genes and Cluster 2 significantly different genes.

In addition, we analysed key gene modules closely associated with the CRGcluster using the WGCNA algorithm. We constructed a scale-free network using β = 13, R2 = 0.9 as a criterion (**Figure 8A**). 5413 genes were classified into 10 key modules and the heat map depicted the TOM of all module-associated genes (**Figures 8B–D**). Analysis of module-clinical characteristics (Cluster1 and Cluster2) relationships showed that the MEturquoise module (931 genes) had the highest correlation with Cluster2 (0.65) and high intra-module gene significance (**Figure 8E**). The MEturquoise module gene correlation analysis with Cluster 2 is shown in **Figure 8F**.

### 3.6 Construction and evaluation of machine learning models

**Figure 9:**
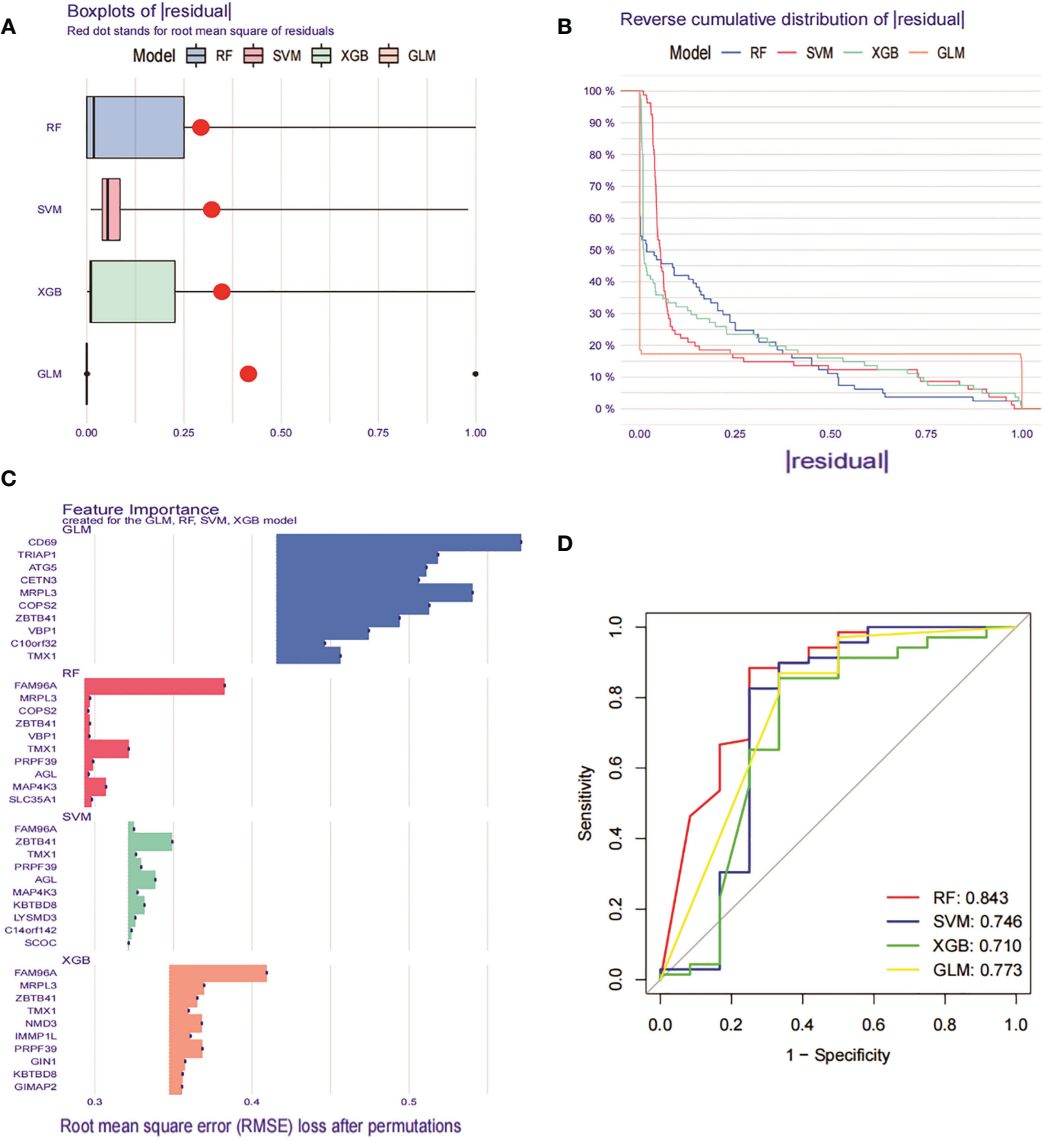
Construction and evaluation of RF, SVM, GLM and XGB machine models. **(A)** Box plot of residuals for the four machine learning models. The red dots represent the root mean square of residuals (RMSE). **(B)** Cumulative residual distribution of the 4 machine learning models. **(C)** Significant functions of the 4 machine learning models. **(D)** ROC curves of the 4 machine learning models plotted based on a 5-fold cross-validation of the test set.

To further identify specific genes with high diagnostic value, we built four machine learning models, namely RF, SVM, GLM and XGB, based on the 276 DEGs in GSE93272 with crossover genes in the MEturquoise module hub gene. using the “DALEX “ package was used to compare the above four models and to analyse the residual distribution of each model. The results show that the RF model has the relatively lowest residuals (**Figures 9A, B**). We ranked the top 10 significant genes of each model based on Root mean square error (RMSE) (**Figure 9C**). The ROC curves of the four models were then plotted based on the 5-fold cross-validation to comprehensively assess their discrimination performance. The AUCs of the four models were, in order, RF: AUC = 0.843; SVM, AUC = 0.746; XGB, AUC = 0.710; and GLM, AUC = 0.773 (**Figure 9D**). Taken together, we concluded that the RF model was able to better differentiate between the different clusters of patients. the RF model ultimately obtained five significant genes (SLC35A1, PRPF39, MAP4K3, TMX1 and FAM96A), which were used as predictive genes for subsequent analysis.

**Figure 10:**
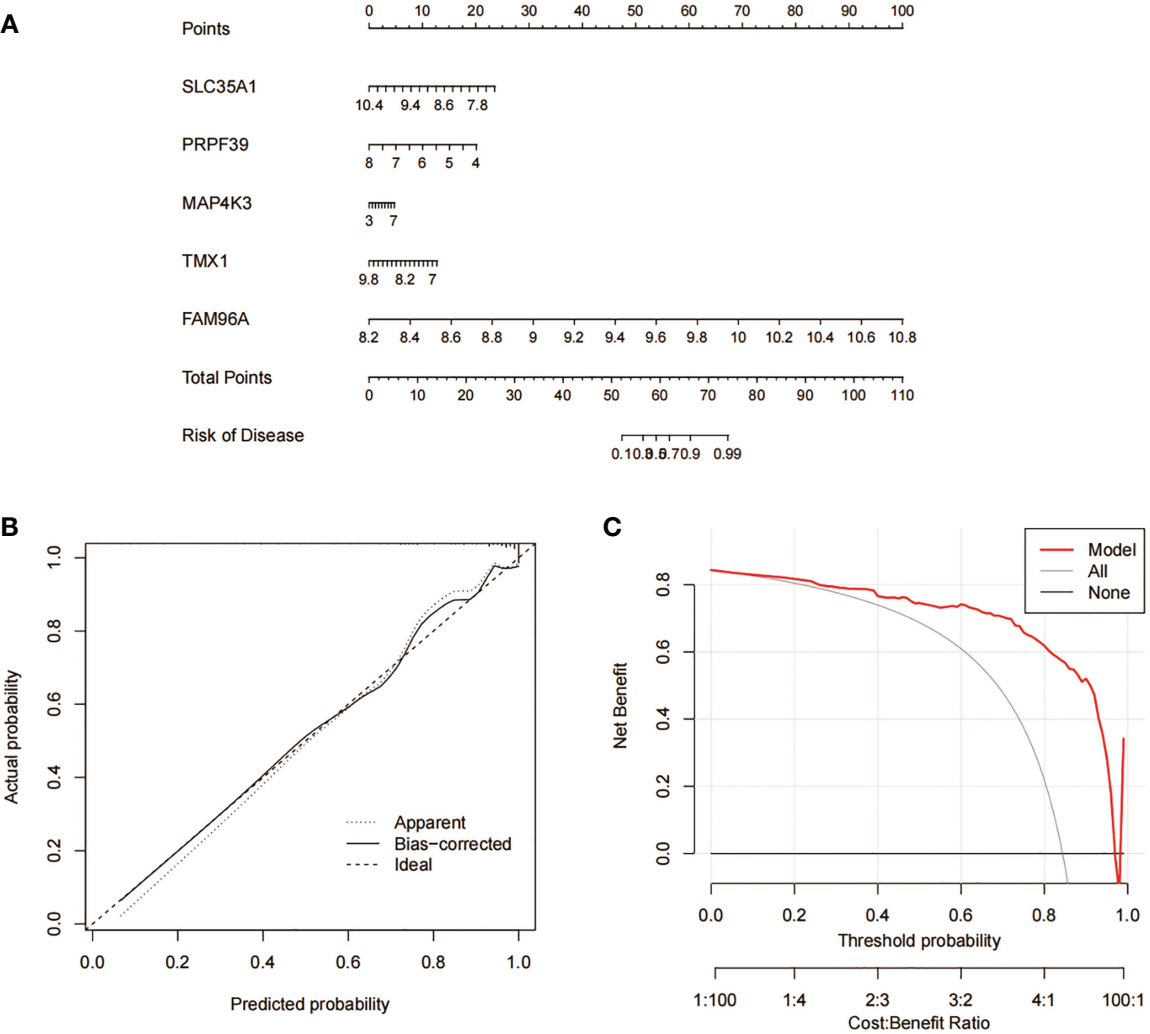
Validation analysis of RF predicted genes. **(A)** Nomogram model for predicting RA risk based on 5 genes in RF. **(B)** Construction of calibration curve. **(C)** Construction of DCA.

We constructed column line plots to further evaluate the predictive effectiveness of the RF model (**Figure 10A**). The predictive efficiency of the constructed line plot model was evaluated using a combination of the calibration curve and DCA, with the calibration curve showing a small margin of error between the actual and predicted risk of RA clustering (**Figure 10B**), and the DCA results indicating that the line plot is highly accurate and can provide some reference and basis for clinical treatment decisions (**Figure 10C**).

### 3.7 RF candidate gene evaluation analysis

**Figure 11:**
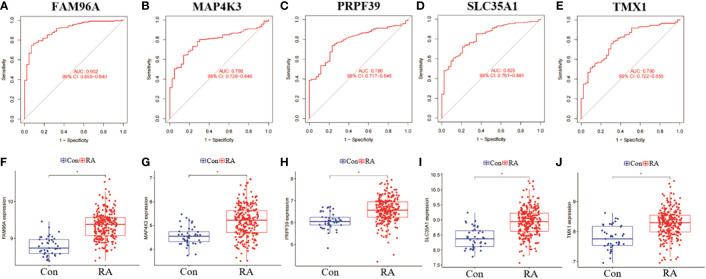
Validation and expression analysis of RF predicted genes. **(A-E)** ROC plots of RF 5 genes. **(B-J)** Differences in expression levels of RF 5 genes between RA and non-RA. *p< 0.0001.

In addition, we analysed the ROC curves of five genes, SLC35A1, PRPF39, MAP4K3, TMX1 and FAM96A, between RA and non-RA patient samples, and also compared their differential expression levels in different samples. the results of the ROC curves showed that FAM96A had the highest diagnostic value (AUC=0.902), while the other four genes had AUC all over 0.750, which was a more satisfactory prediction (**Figures 11A–E**). In addition, by performing a differential analysis of their expression levels, the results showed that all five genes were significantly more expressed among RA patients than in the non-RA patient group (**Figures 11F–J**).

### 3.8 Predictive gene validation by qRT-PCR in experimental RA animal models

**Figure 12:**
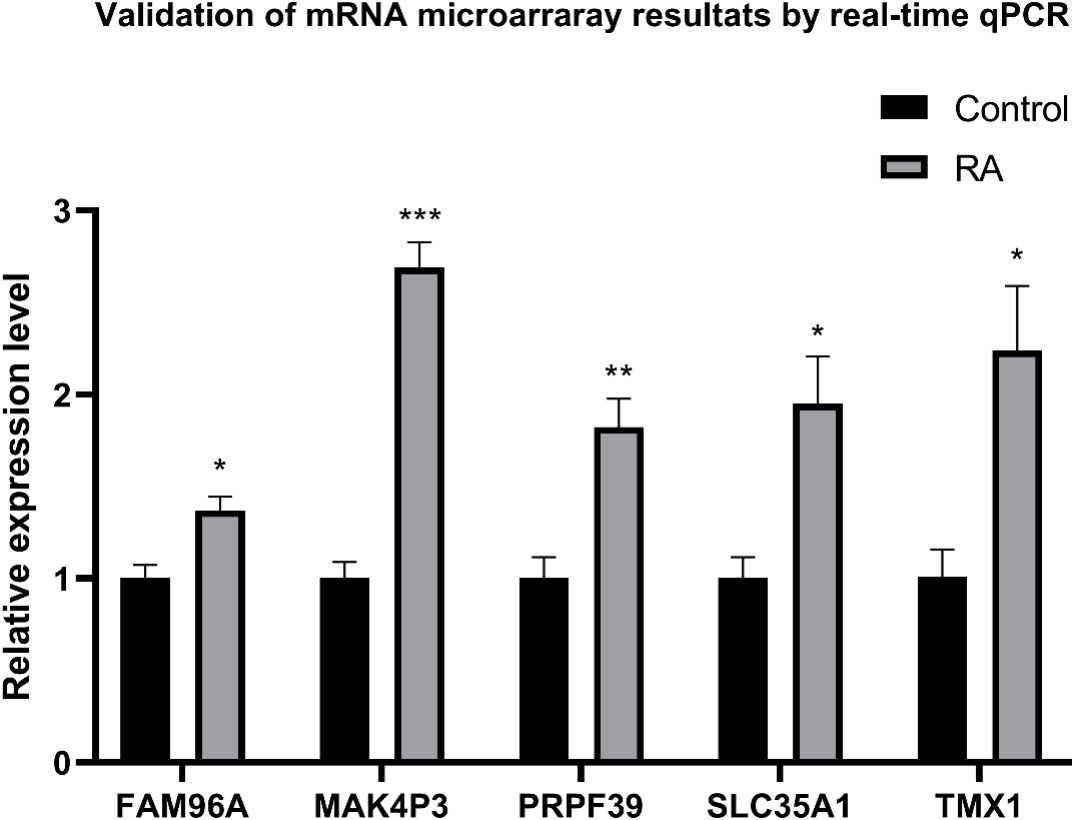
qRT-PCR was used to express ankle genes in RA models and controls. The expressions of 9 genes in the ankle joint of the RA model, including FAM96A, MAK4P3, PRPF39, SLC35A1 and TMX1, were significantly increased. *P<0.05, **P<0.01, ***P<0.001.

qRT-PCR was used to detect and compare the expression of identified genes in the ankle tissue of the RA model and control rats (**Figure 12**). Compared with normal controls, five genes were significantly increased in the ankle RA rat model, including FAM96A, MAK4P3, PRPF39, SLC35A1, and TMX1(P<0.05).

## 4 Discussion

Because the complex pathophysiological mechanisms of RA are not fully understood, early and accurate diagnosis as well as treatment and management of RA is essential (41). Over the past few decades, there have been increasing advances in the symptomatic treatment of RA (NSAIDs and GCs) and the management of disease remission (DMARDs) (4). The identification of more appropriate molecular clusters is therefore essential to guide the individualised treatment of RA. The recently reported copper-dependent form of cell death is mainly caused by aggregation of lipid acylation-related proteins, loss of iron-sulfur cluster proteins and a series of other stress responses culminating in cell death, and the association with RA is multifaceted (15, 23). However, the specific mechanisms and roles of copper death regulation in various diseases have not been further investigated. Therefore, this study attempts to elucidate the relationship between copper death-associated genes and RA phenotypes, as well as to analyse their specific role in the immune microenvironment, and we also use copper death-associated genes to predict subtypes of RA. Finally, the expression of the identified predicted genes was detected and compared in the ankle tissue of the RA model and control rats by qRT-PCR. Five genes were significantly increased in the ankle RA rat model compared to the normal control group.

In the present study, we conducted the first comprehensive analysis of the differential expression of CRGs between healthy and RA patients. The results showed that 7 out of 12 CRGs were differentially expressed between RA and healthy individuals and that the expression of 7 CRGs was significantly higher in RA patients than in normal individuals, suggesting that CRGs may be closely associated with the development of RA. Subsequently, we analysed the correlation between CRGs to elaborate on the association between CRGs and RA. In contrast, RA patients had significant infiltration of B cells memory, T cells CD8, T cells gamma delta, Macrophages M0, Macrophages M1, Macrophages M2, Dendritic cells activated, Neutrophils. In addition, we performed an unsupervised clustering analysis based on CRG expression levels and identified two clusters of copper death-related gene molecules. Four differentially expressed CRGs, namely FDX1, DLD, LIPT1, and LIAS were expressed at significantly higher levels in CRGcluster C2 than in CRGcluster C1 and had higher levels of immune infiltration in T cells CD8, T cells CD4 memory activated, and T cells gamma delta. We further grouped RA patients by re-clustering the intersecting genes between CRGcluster C1 and CRGcluster C2 and obtained two different subgroups: genecluster C1 and genecluster C2. genecluster C2 showed that the expression levels of LIPT1, FDX1, DLD, LIAS genecluster C2 showed significantly higher levels of LIPT1, FDX1, DLD and LIAS than genecluster C1. genecluster C2 showed higher levels of T cell CD4 memory activated and T cell gamma delta. The correlation between genecluster C2 and RA may be higher than that between genecluster C1. copper death-related scores showed that CRGcluster 2 and genecluster C2 had higher scores than CRGcluster C1 and genecluster C1, respectively, and the expression levels of 12 CRGs were also higher than those of CRGcluster C1, and genecluster C1.

In recent years, machine learning (ML) has been widely used in the clinical field and is considered an important tool in healthcare (42, 43). ML has also contributed to the prediction of biomarkers for RA disease, prognostic modelling, and drug screening. And ML has also contributed to the prediction of RA disease biomarkers, the development of prognostic models, and drug screening (44, 45). In comparison with the traditional statistical model, the comprehensive analysis of ML ensures the robustness of the model and improves the prediction accuracy through iterative algorithm (46, 47). In the current study, we compared the predictive performance of four machine learning models (RF, SVM, GLM and XGB). These models are based on 276 cross genes in MEturquoise modules closely related to DEGs and CRGclusters in GSE93272 and established a predictive model based on RF, which gives the best prediction (AUC = 0.843). We subsequently identified five significant predictive genes (SLC35A1, PRPF39, MAP4K3, TMX1 and FAM96A) based on RF. Solute carrier family 35 member A1 (Slc35a1) is a specific transporter protein. In sialic acid (SA) metabolism, the transfer of cytidine-5’-monophosphate-SA to the medial and trans-Golgi is a substrate for the sialylation of proteins by various sialic acid transferases (48). SA is a highly diverse family of acidic glycans, whose functions are to stabilize cell membranes, facilitate interactions with the environment, enhance intercellular adhesion and signalling, and regulate the affinity of ligands for receptors (49, 50). Previous studies have found that Slc35a1 is a key step in all SA synthesis pathways, and that ablation of Slc35a1 decreases SA translocation to the cell surface and thus reduces expression (51–53). On the other hand, SA-modified liposomal formulation, based on the high expression of L-selectin in peripheral blood neutrophils and SA as its targeting ligand have been proved to be an effective neutrophil-mediated drug delivery system targeting RA (54).

Pre-mRNA splicing factor 39 (PRPF39) is an alternative splicing factor. It is a homolog of the yeast Prp39 and Prp42 paralogs, that is tightly coupled to gene transcription and subsequent splicing processes (55, 56). Previous studies have found that PRPF39 expression levels strongly influence *in vitro* splicing (57), particularly in immune cell differentiation and activation, for which regulated intron retention has been shown to play an important role in controlling gene expression and function (58). On the other hand, it was found that RA was closely related to splicing variants (59, 60), and by examining the altered levels of splicing mechanism components and inflammatory mediators, it was found that the dysregulation of splicing mechanism components, such as SNRNP70, SNRNP200 and U2AF2, could be reversed when TNFi was intervened *in vivo (* 61). Previous studies have identified an alternatively spliced TNRF2 isoform, a soluble receptor. The alternatively spliced absence of exons 7 and 8 (DS-TNFR2), which encode transmembrane and cytoplasmic structural domains, constitutes the majority of sTNFR2 in RA patients and serum sTNFR2 is strongly associated with RA activity and severity (62–64).

Mitogen-activated Protein kinase kinases (Also called Germinal center kinase-like kinase, GLK/MAP4Ks), belong to the mammalian Ste20-like family of serine/threonine kinases (65). Previous studies have found that MAP4K3 is involved in extracellular signalling to regulate gene transcription, apoptosis and immune inflammation (66–68); On the other hand, MAP4K3 also activates mTOR signalling in epithelial cell lines after sensing cellular nutrient and energy levels and plays a key role in activating NF-κb signalling in T cells after antigen stimulation (69). Previous studies have demonstrated that MAP4K3 expression levels are significantly increased in peripheral blood T cells from patients with autoimmune diseases such as RA (70), SLE (71) and adult-onset Still’s disease (66). In a study by Chen et al., it was found that the frequency of overexpression of MAK4P3 by circulating T cells was significantly increased, and the production of MAK4P3 was found to decrease in parallel with disease remission in these patients, thus suggesting that MAK4P3 is closely related to immune like disease activities (66, 71). These enrich the evidence that overexpression of MAK4P3 is associated with a series of inflammatory diseases. In conclusion, MAP4K3 plays an important role in immune cell signalling, immune response and inflammation.

ER is known to be the site of biosynthesis of all secreted and membrane proteins. Its inner lumen is a unique environment that is critical for the correct folding of proteins secreted or displayed on the cell surfacee (72). ER stress, in turn, results from the accumulation of unfolded proteins (UPR) in the ER and can cause a range of pathologies such as chronic autoimmune inflammation (73). Previous studies have found that RA inflammation and ER stress work in parallel by driving inflammatory cells to release cytokines that induce chronic ER stress pathways, and synovial cells promote inflammation by continuously producing large amounts of proteins and that ERAD may be a necessary processing system for ER homeostasis in order to prevent further development (74, 75); Another study has shown that enhanced ERAD can effectively remove unfolded proteins from the ER, thereby indirectly inhibiting UPR activation (76). This suggests that a dysregulated ER response is closely linked to the development of synovial hyperplasia and chronic arthritis. TMX1 is a topology-specific endoplasmic reticulum-resident reductase, the most characteristic member of the TMX family, whose main biological functions are protein folding and ER-associated degradation Ca^2+^ flux regulation (77, 78). Thus TMX1 may be closely associated with RA by promoting misfolded polypeptide mismatches across the ER membrane for ER-related degradation.

Family with sequence similarity 96 member A (FAM96A), also known as Cytosolic iron-sulphur (Fe/S) assembly component 2A (CIAO2A), is an evolutionarily conserved protein highly expressed in the immune system, associated with cytosolic iron assembly and tumour suppression, and is widely expressed in many tissues (79, 80). Yin et al. found that the release of relevant pro-inflammatory factors was significantly slowed in FAM96A knockout mice and that sepsis could be slowed by the action of macrophages (79). Probably due to the important role of macrophages in immune homeostasis and inflammatory processes (81, 82), FAM96A may regulate RA by controlling the secretion of multiple inflammatory factors as well as multiple metabolisms of macrophages.

However, there are still some limitations in this study. First, our current study is based on bioinformatic analysis and simple experimental validation. In the future, more comprehensive clinical or experimental studies are needed to validate the relationship between copper death-related candidate genes and RA. In addition, more detailed clinical characterisation is needed to test the performance of the prediction model, and more RA samples are needed to assess the accuracy of copper death-related candidate genes.

## 5 Conclusion

In this study, we constructed column line plots based on 5 genes, SLC35A1, PRPF39, MAP4K3, TMX1 and FAM96A, and the results showed that the predictive effect of the model was more obvious. In addition, we analysed the expression differences of the above five genes between RA and non-RA patients, and the results showed that the expression levels of all five genes were significantly higher among RA patients than the non-RA patient group; and the ROC curve results showed that FAM96A had the highest diagnostic value (AUC=0.902). Finally, validation was performed by qRT-PCR assay. In conclusion, the five important candidate genes obtained based on the RF model have more satisfactory results in assessing the pathological outcomes and subtypes in RA patients.

## Data availability statement

The datasets presented in this study can be found in online repositories. The names of the repository/repositories and accession number(s) can be found in the article/supplementary material.

## Ethics statement

The animal study was reviewed and approved by Changchun University of Chinese Medicine Scientific Research Ethics Review Committee No. ccucm-2017-0015.

## Author contributions

YZ, XL, SS and CL designed the study. XL, LN, QZ and JH collected the literature and WS, JZ verified the results through experiments. YZ wrote the article. CL and SS reviewed and edited the manuscript. CL and SS contributed equally to this work. All authors have read and approved the final manuscript. All authors contributed toward data analysis, drafting and critically revising the paper and agree to be accountable for all aspects of the work.
