# Supplementary figures and images for: Identification of copper death-associated molecular clusters and immunological profiles in rheumatoid arthritis
Source: Front Immunol. 2023 Feb 20;14:1103509. doi: 10.3389/fimmu.2023.1103509 (PMC9986609; doi:10.3389/fimmu.2023.1103509)

Supplementary material 1.

The result of the ConsensusClusterPlus (k=3 and 4).


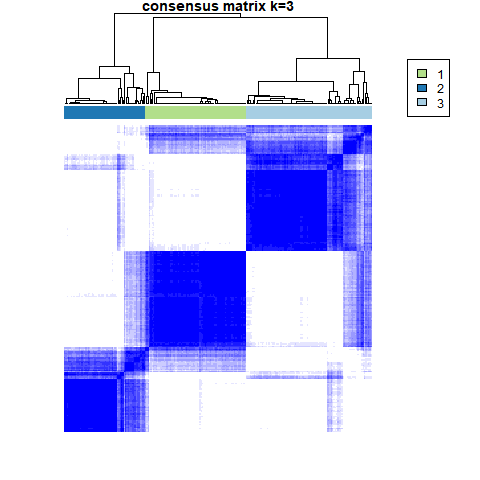


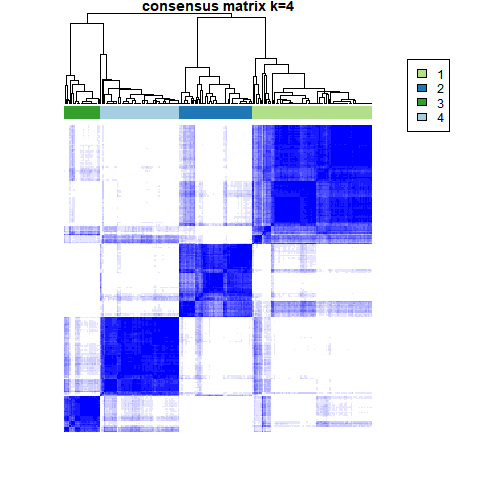

Supplement: Supplementary file 1 [file DataSheet_1.docx]
